# Understanding *Ligilactobacillus salivarius* from Probiotic Properties to Omics Technology: A Review

**DOI:** 10.3390/foods13060895

**Published:** 2024-03-15

**Authors:** Yong Yang, Xin Song, Guangqiang Wang, Yongjun Xia, Zhiqiang Xiong, Lianzhong Ai

**Affiliations:** Shanghai Engineering Research Center of Food Microbiology, University of Shanghai for Science and Technology, Shanghai 200093, China; wy931221@sina.com (Y.Y.); daohongxuan@126.com (X.S.); 1015wanggq@163.com (G.W.); dreamup@126.com (Y.X.); xiongzq@hotmail.com (Z.X.)

**Keywords:** *Ligilactobacillus salivarius*, probiotic, functional properties, omics technologies, gut microbiota

## Abstract

*Ligilactobacillus salivarius* (basonym: *Lactobacillus salivarius*, *L. salivarius*) is a type of lactic acid bacteria (LAB) commonly found in the oropharyngeal-gastrointestinal tract (OGT). It has gained significant attention due to its probiotic and functional properties as well as its various health-promoting roles. *L. salivarius* strains exhibit strong resistance and adhesion in the OGT along with outstanding antioxidant and antimicrobial properties. Additionally, numerous *L. salivarius* strains have the ability to produce bacteriocins with antagonistic activity. These probiotic characteristics of *L. salivarius* indicate its remarkable potential in promoting favorable effects on human health. It has also been observed that *L. salivarius* has a positive effect on the composition of intestinal microbiota, thereby improving the metabolic profiling of intestinal microbiota, promoting a healthy and balanced internal environment. In recent years, multi-omics technologies such as genomics, transcriptomics, proteomics and metabolomics have been employed to gain a deeper understanding of the roles and mechanisms of *L. salivarius* associated with its functional properties. This review aims to provide an overview of the probiotic characteristics of *L. salivarius*, containing its specific interactions with the host microflora, as well as insights from omics studies.

## 1. Introduction

*Ligilactobacillus salivarius*, formerly named *Lactobacillus salivarius*, has been referred to as this species name for almost 70 years. It was initially described by Rogosa et al. in 1953 as an obligatory homo-fermentative lactic acid bacteria [1,2]. Over time, *L. salivarius* has undergone several reclassifications and is now recognized as a Gram-positive bacterium capable of both homotypic and parthenogenic heterotypic fermentation [3]. In the past three years, *L. salivarius* strains have been reclassified and moved from the *Lactobacillus* genus to a new genus called *Ligilactobacillus*, which consists of 16 species. The name *Ligilactobacillus* implies a combination, with a host-adapted lifestyle, specifically referring to the vertebrate host of *L. salivarius* [4].

*L. salivarius* is a non-motile, non-sporulating, oxidase- and catalase-negative, rod-shaped microorganism with a general size of 0.6–1.9 μm × 1.5–5 μm. *L. salivarius* exists in different niches and currently isolates are primarily derived from the intestines or feces of birds and mammals [5], e.g., geese, chickens, turkeys, pigeons, ducks, pigs and cattle. Moreover, it has also been shown to exist in the oral cavity, vagina and breast milk of humans, and in honeybee guts, and in foods such as grape wine, meat and St. Ivel cheese [5]. Recently, *L. salivarius* has received increasing attention as a potential probiotic, and various applications of *L. salivarius* strains have been explored.

Based on available data as of April 2023, the average genome size of *L. salivarius* strains that have been sequenced is 1.99 ± 0.14 Mbp, containing 1946.70 ± 382.15 genes with a GC content of 32.79 ± 0.16%. In addition to chromosomes, the genome typically includes a *repA*-type megaplasmid ranging from 100 to 380 kbp and small plasmids. Studies have shown significant differences in both chromosomal and plasmid sequences among *L. salivarius* strains, particularly in genes encoding glycoside hydrolases (GH), bacteriocins, proteases and EPS synthesis.

In terms of functional characteristics, most *L. salivarius* strains have a strong tolerance to acidic pH, resistance to the OGT conditions and adhesion to the intestinal mucosa, enabling them to effectively colonize in the host. In addition, excellent antioxidant and antibacterial properties have also demonstrated that this ligilactobacillus can have favorable effects on the host health. *L. salivarius* strains possess antagonistic properties against bacterial pathogens such as *Salmonella enterica* (*S. enterica*), *Clostridium perfringens* (*C. perfringens*), *Staphylococcus aureus* (*S. aureus*), *Klebsiella pneumoniae* (*K. pneumoniae*), *Pasteurella multocida* (*P. multocida*), *Riemerella anatipestifer* (*R. anatipestifer*) and *Campylobacter* sp. This ability is attributed to the production of lactic acid, H_2_O_2_ and bacteriocins, as well as the capacity to colonize the gut for an extended period, leading to the exclusion of unfavorable microflora [6]. Moreover, previous studies have shown that most *L. salivarius* strains can improve the composition of the intestinal microflora. Indeed, the correlation between *L. salivarius* strains and indigenous gut microbiota has emerged as a popular area in scientific research. On the other hand, studies have shown that *L. salivarius* strains possess several functional properties that are beneficial to the food industry. These properties include improving nutritional quality, enhancing flavor properties, exhibiting antioxidant and antimicrobial activities, increasing the shelf-life of foods, and reducing undesirable compounds [7]. Based on these favorable functional characteristics mentioned above, *L. salivarius* has been awarded the “generally recognized as safe” (GRAS) status by the United States Food and Drug Administration (FDA), and listed as a recommended biological agent intentionally added to food by the European Food Safety Authority (EFSA) and the Ministry of Public Health of China [8]. While studying the functional properties of *L. salivarius*, the multi-omics technologies including genomics, transcriptomics, proteomics and metabolomics have achieved rapid development. Among them, genomics and transcriptomics provide a strong guarantee for genetic information analysis and the functional gene identification of probiotics. Proteomics and metabolomics are very effective methods to study the adaptive mechanism of probiotics to physiological and environmental changes. In a recent study, Lugli et al. proposed a novel concept of “probiotic genomics”, which undoubtedly provides a proprietary identity card for each probiotic [9]. Therefore, omics technologies are growing in significance as their ability to elucidate the molecular mechanisms underlying the functional and probiotic properties of LAB. This review summarized the probiotics properties of *L. salivarius* as well as the application of omics approaches in *L. salivarius* strains. It provides a reference for subsequent research applications of *L. salivarius* strains.

## 2. Probiotic Properties and Roles of *Ligilactobacillus salivarius*

*L. salivarius* is an important member of the LAB family and it has been widely used as a probiotic due to its excellent characteristics (Table 1). Although early studies focused on the isolation of probiotic strains of *L. salivarius* and their bioactive metabolites (mainly bacteriocins), more research is now available to better understand the role of *L. salivarius* strains and their metabolites in various fields and their adaptability to environmental stresses.

### 2.1. Resistance to Oropharyngeal-Gastrointestinal Conditions

A requisite condition for any microorganism to be a probiotic is the ability to survive or pass the harsh conditions of the GT. The stomach is a very unfriendly environment, with its internal gastric juice consisting mainly of pepsin and hydrochloric acid (HCl). This directly contributes to the low pH (1.5–4.5) of the gastric environment. Therefore, acidic pH tolerance is considered to be the main criterion for probiotic screening. In this unfavorable environment, *L. salivarius* strains are potential probiotics by maintaining intracellular pH balancing, ATR signaling pathway and macromolecule protection and repair, among other strategies to achieve pH and bile tolerance [42]. In addition, the acid resistance of some LAB can also be improved by exposing themselves to non-lethal acidic conditions through acid resistance reactions [43]. It is known that different strains of the same LAB species have highly variable acid resistance and this is also true for *L. salivarius* strains. A study shows that *L. salivarius* IBB3154 showed good resistance and survived at low pH (value is 3.5) conditions [44]. Similarly, Sajedinejad et al. demonstrated that *L. salivarius* NK02 displayed significant tolerance to low pH conditions. Specifically, this strain exhibited survival rates (7 logs CFU/mL) when exposed to simulated gastric juice (pH 2.2). Furthermore, *L. salivarius* NK02 was also found to be significantly tolerant to bile, lysozyme, and 1–5% NaCl [37].

In addition to gastric acid, probiotics must also tolerate exposure to bile salts in the small intestine. Bile salt is a sodium or potassium salt formed by the combination of bile acids secreted by hepatocytes with glycine or taurine. It is the main component of bile involved in fat digestion and absorption. Cell membrane disruption, DNA damage, intracellular acidification, oxidative stress and metabolic changes triggered by bile salts, pose a serious threat to the colonization and survival of probiotics [45]. Therefore, bile tolerance is another major criterion for screening potential probiotic strains. *L. salivarius* strains, an OGT natural flora, have been exposed to bile salt stress for a long time and have evolved various mechanisms to cope with bile salt toxicity, mainly including the production of bile salt hydrolases (BSHs), alteration of cell membrane composition and structure, and the use of a transport system to transfer bile salts [46]. BSH is considered to be the main component of bile tolerance in LAB. It is responsible for catalyzing the depolymerization of glycine and taurine residues in cholesterol. Although the exact nature of how bile salt depolymerization limits negative effects on cellular homeostasis is unknown, many literatures have reported that bile salt hydrolase expression is associated with bile resistance in many lactobacilli species [47]. However, Fang et al. demonstrated that BSH is not the primary determinant of bile resistance in *L. salivarius* strains, and may have additional biological importance because of its varying effects upon bile as a signaling molecule in the host [48]. Pan et al. also showed that there was no association between bile salt hydrolase and bile salt tolerance in *L. salivarius* strains and indicated that bile salt tolerance in *L. salivarius* strains was associated with processes such as peptidoglycan synthesis, the phosphotransferase system (PTS) and DNA damage repair [49]. This is consistent with the results of Wang et al. [50] and Lv et al. [51].

In addition to gastrointestinal microbiota, oral microbiota is also part of the human microbiome. In the daily diet process, it is essential to add salt, vinegar and other food ingredients to various diets, and these high osmotic pressure foods will inevitably affect the survival and growth of lactic acid bacteria in the oral tract. Therefore, probiotics must also tolerate a variety of complex conditions. *L. salivarius* AR809 was isolated from a healthy adult oral cavity with good resistance to acidic pH, bile, lysozyme and H_2_O_2_ [35,42].

It can be seen that most of the *L. salivarius* have the characteristics of acid and bile salt tolerance. Certainly, tolerance to various conditions could impact the probiotic potential of different strains of *L. salivarius*. Nevertheless, on the other hand, it is essential to recognize that resistance to low pH and bile is crucial for probiotics, but it does not guarantee that acid pH-tolerant and bile-tolerant strains will exhibit probiotic properties.

### 2.2. Adherence to the Intestinal Mucosa and Extracellular Matrix Components

**Figure 1:**
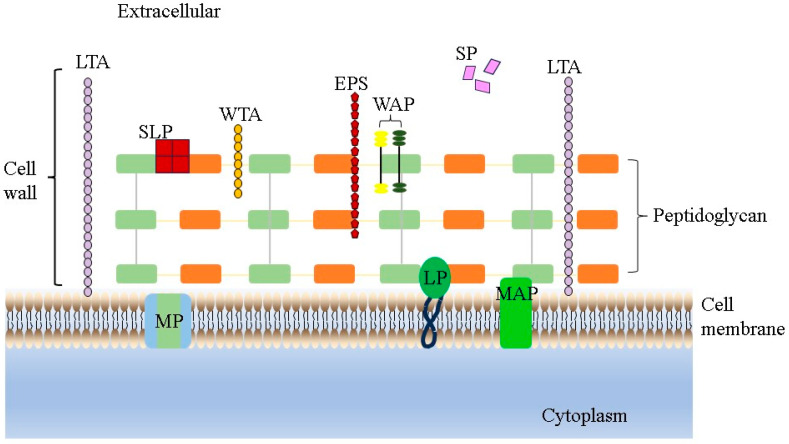
LAB cell wall structure. LTA represents lipoteichoic acids, WTA represents wall teichoic acids, EPS represents exopolysaccharides, WAP represents wall-associated proteins, SLP represents surface layer proteins, SP represents secreted proteins, MP represents membrane proteins, LP represents lipoproteins and MAP represents membrane-associated proteins.

Adhesion is an important prerequisite for the colonization and function of LAB strains in the OGT, which is directly related to cell wall components such as adhesins, polysaccharides and proteins (Figure 1). It includes two steps, non-specific adhesion and specific adhesion. Through adhesion, LAB can be permanently colonized in the IEC membrane, enhance the signal exchange between cells, promote the stability of intestinal flora, regulate the body’s immunity, form a biological barrier, and resist pathogenic bacteria [52]. Therefore, adhesion is also one of the important criteria for screening probiotic lactic acid bacteria in vitro. In this case, many researchers have begun to study the adhesion ability of *L. salivarius* strains. A study conducted by Jia et al. [35] demonstrated that in a cell adhesion assay simulating the human oral environment, the extent of adhesion to FaDu cells of *L. salivarius* AR809 (31.1%) was significantly higher than that of *L. plantarum* AR113 (4.44%) and *L. plantarum* AR195 (8.28%). In addition, *L. salivarius* AR809 also significantly reduced the adhesion effect of *S. aureus* to FaDu cells through exclusion, competition and displacement. In a similar way, Dash et al. demonstrated that the *L. salivarius* F14 strain had good adhesion to the Caco-2 cells through the co-culture model in vitro, and it also could significantly inhibit the adhesion process of *S. typhimurium* ST-Xen 33 on Caco-2 cells [10]. For their part, Zhang et al. [53] and Ren et al. [54] showed that *L. salivarius* strains had good adhesion to the Caco-2 cells which could be associated with the presence of genes that encode different proteins attributed to adhesion to different extracellular matrices and intestinal mucus [46]. Of course, we should also list more in vivo results to confirm that *L. salivarius* strains can adhere to the intestinal mucosa/extracellular matrix components and persist.

### 2.3. Antioxidant Activity

Oxidative stress is when the balance between anti-oxidants and prooxidants in the cell is disturbed, resulting in DNA hydroxylation, protein denaturation, lipid peroxidation and ultimately cell apoptosis. It is the fundamental reason for aging and aging-related diseases, which can induce diabetes, atherosclerosis, arthritis, hyperlipidemia, cardiovascular diseases and many other diseases [55]. In recent years, a large number of studies have shown that lactic acid bacteria can remove the active oxygen molecules in the intestine, so that the active oxygen molecules in the body remain relatively stable, thereby reducing the body damage caused by the oxidation reaction. These lactobacilli exert antioxidant effects mainly through scavenging reactive oxygen radicals in and around cells, chelating metal ions, alleviating lipid peroxidation, modulating their antioxidant defense system, regulating the host cell antioxidant defense system, and modulating host cell antioxidant-related signaling pathways [56]. With the gradual increase in reports on the antioxidant effect of lactobacillus, the understanding of the antioxidant function of lactobacillus is also increasing. Zhang et al. identified a strain of *Limosilactobacillus fermentum* YLF016, which contained various antioxidant enzyme encoding genes, as well as having a strong ability to scavenge 2,2-diphenyl-1-picrylhydrazyl (DPPH) and hydroxyl radicals (-OH) scavenging ability [57]. Zhai et al. evaluated the antioxidant activity of 10 lactobacillus strains and found that *L. plantarum* CCFM 8661 exhibited the strongest lipid peroxidation inhibitory activity [58].

Currently, there have been studies on the *in vivo*/*vitro* antioxidant function evaluation of *L. salivarius* strains at home and abroad. Zhang et al. studied the protective mechanism of *L. salivarius* by establishing an alcohol injury model [59]. Specifically, *L. salivarius* M18-6 protected mouse hepatocytes from alcohol-induced oxidative stress damage by downregulating serum alanine transaminase (ALT) levels, upregulating superoxide dismutase (SOD) levels and activating the keap1-Nrf2 signaling pathway. This research provided a scientific basis for the clinical application and product development of the *L. salivarius* strain for alcohol injury. Wang et al. revealed the molecular mechanism of *L. salivarius* Ren in response to bile-induced oxidative stress by transcriptomics and proteomics [60]. *L. salivarius* MG242 isolated from the human vagina by Kang et al. exhibited good antioxidant properties with DPPH radical scavenging rate and 2, 2′-azino-bis (3-ethylbenzothiazoline-6-sulfonic acid) (ABTS) of 56.9% and 97.1%, respectively [61]. Chooruk et al. investigated the antioxidant activities of 201 LAB strains in vitro [62]. Among them, the antioxidant capacity of *L. salivarius* differed significantly from other lactobacillus, which also indicates the specificity of antioxidant properties of different species and strains. The supplementation of *L. salivarius* AP-32 in Parkinsonian rats modulates short-chain fatty acid (SCFA) production, increases antioxidant enzyme activity and protects mitochondria from reactive oxygen species (ROS)-induced damage [63]. Therefore, the antioxidant potential of *L. salivarius* can provide ideas for anti-aging related research.

### 2.4. Active Metabolites and Its Antimicrobial Activity

**Figure 2:**
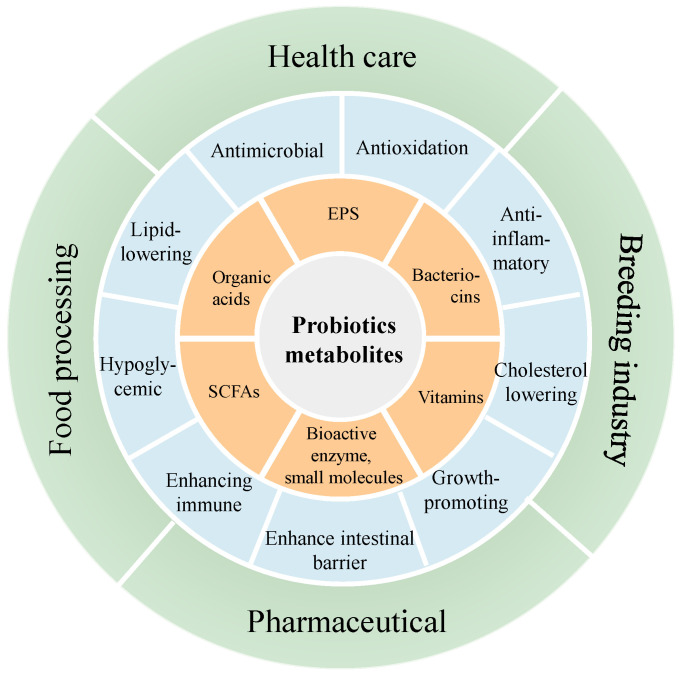
Active metabolites of probiotics, their functions and applications.

Although the traditional view is that only live probiotics can exert their probiotic effects, more and more studies have shown that some of the probiotic properties of probiotics are closely related to the active products produced by their metabolism. At present, the active metabolites of probiotics mainly include EPS, bacteriocins, organic acids, SCFAs, vitamins and some bioactive enzymes and small molecules (Figure 2). These active metabolites have been proven to have anti-inflammatory, antimicrobial, antioxidation, immune regulating properties useful for the prevention or treatment of a variety of metabolic diseases. Antimicrobial properties are one of the most notable characteristics of probiotics. This can be achieved through competition for nutrients and adhesion space, inducing environments that are harmful to pathogens, and that produce antimicrobial metabolites and regulate immune responses [46]. This subsection is devoted to the antimicrobial activity of *L. salivarius* strains.

*L. salivarius* strains can produce several antimicrobial active compounds that exhibit antagonistic activity against pathogenic organisms. First, many *L. salivarius* strains are good producers of many small proteins such as antimicrobial peptides and bacteriocins. This allows them to compete with other bacteria in the environment and disrupt the cell walls and cell membranes of pathogenic bacteria, causing the intracellular material to leach out, thus producing an antibacterial effect [64,65,66,67]. *L. salivarius* PS7 showed strong antagonistic activity against ten acute otitis media (AOM) related pathogens (*Streptococcus pneumoniae* MP07, *Streptococcus pyogenes* MP03, *S. aureus* MP29, *Staphylococcus epidermidis* MP33, *Alloiococcus otitidis* MP02, *Enterococcus faecalis* MP64, *Haemophilus influenzae* MP04, *Moraxella catarrhalis* MP08, *P. aeruginosa* MP24 and *E. coli* MP69) [28]. Similarly, Martín et al. found that *L. salivarius* CECT5713 inhibited the growth of Gram-negative bacteria (including, *E. coli* CECT4076, *K. pneumoniae* CECT 142, *K. oxytoca* CECT 860T and *Proteus vulgaris* CECT484), Gram-positive bacteria (*E. faecium* P21, *E. faecalis* TAB28, *Listeria monocytogenes* ScottA, *L. monocytogenes* Ohio, *L. innocua* RdC, *S. aureus* CECT5191, *S. epidermidis* CECT 231, *Lactococcus lactis* MG1614 and *Latilactobacillus sakei* NCFB2714) and the yeasts *Rhodotorula mucilaginosa* CECT10359, this effect being greater in *L. monocytogenes* Ohio and *K. oxytoca* CECT 860^T^ [68]. The antimicrobial effect of both strains of *L. salivarius* was attributed not only to the presence of bacteriocins but it was also related to the formation of organic acids (such as common lactic acid and SCFAs) and H_2_O_2_ that leads to a change in the medium, which can alter the development of indicator organisms [69]. Furthermore, *L. salivarius* strains can also produce EPS with natural antibacterial activity, which is usually attributed to its anti-biofilm effect [70]. Bikric et al. found that EPSBIS312 and EPSBIS722 derived from *L. salivarius* BIS312 and *L. salivarius* BIS722, respectively, could significantly inhibit the biofilm formation of *Enter. faecalis* 29212, *Staph. aureus* EB1 and *E. coli* ATCC 11229, and were significantly higher than commercial inulin [71]. This indicates that these two EPS may become substitutes for plant prebiotics (such as inulin) in poultry.

Moreover, many *L. salivarius* strains also possesses antiviral activity. *L. salivarius* YM33 isolated from the feces of nursing piglets has good activity against pig epidemic diarrhea virus (PEDV) and can significantly down-regulate proinflammatory cytokine levels [72]. This research was the first demonstration of the antiviral activity of *L. salivarius* against PEDV. Similarly, Shojadoost et al. reported that *L. salivarius* JTBo9 enhanced the antiviral activity of macrophages of chicken against avian influenza virus infection via virus titer reduction and increased the expression of IL-1β and IFN-γ virus titer in an in vitro cell model [73]. It is also because of the antimicrobial activity of *L. salivarius* strains that it is widely used in the breeding industry. In conclusion, the antimicrobial activity of *L. salivarius* provides favorable conditions for its application in various fields.

### 2.5. Host OGT Micro-Ecosystem Modulation

The host gut is a complex micro-ecosystem consisting of communities of bacteria, viruses, archaea, fungi and protozoa that live in the GT [74], known as the intestinal flora. Gut microbiota performs useful functions including fermenting unused energy substrates, training the immune system with metabolic end products, maintaining the intestinal epithelium, synthesizing host vitamins [75], producing hormones that induce host fat accumulation, metabolizing dietary and pharmaceutical compounds and controlling immune function, and even affects behavior via the gut-brain-axis [76]. 

**Figure 3:**
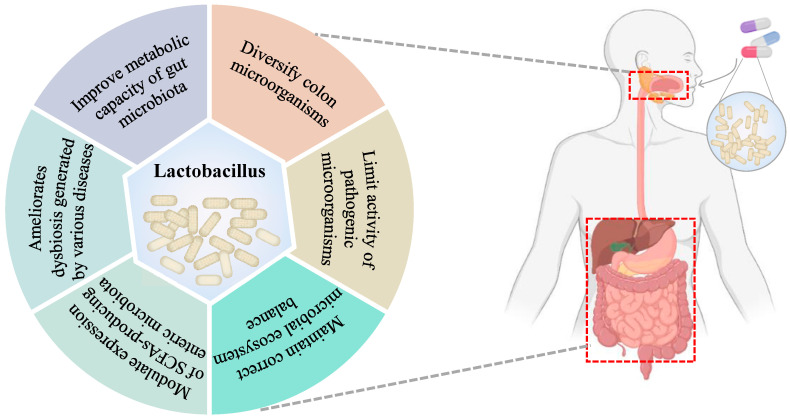
Effects on the gut microbiota following administration of lactobacillus.

Given these facts, intestinal microflora plays a key role in the maintenance of host health and the pathogenesis of many diseases [77]. Therefore, gut microbiota eubiosis is essential for the prevention of infectious and non-infectious diseases and for preventing disturbances (also known as dysbiosis) in the balance of the microbial community equilibrium [78]. However, the gut microbiota is an open micro-ecosystem whose composition and/or activity can be influenced by many elements, including the mode of birth, sex, host inheritance, immune system and host health or disease status, geographic location, social economic factors, diet, the use of therapeutic drugs, etc. [79]. In point of fact, the intestinal microbiota is constantly exposed to transient exogenous microorganisms transmitted through food, as demonstrated by Veiga et al. [80]. In this regard, probiotics can regulate the composition of gut microbiota and correct the abnormal response of the immune system [81], thereby exerting different favorable effects on host health (Figure 3). In summary, probiotics may be a therapeutic strategy to regulate intestinal flora and improve human disease [60,82]. In this sense, several studies have found that different strains of *L. salivarius* have a significant regulatory effect on intestinal flora (Table 2).

For instance, Moturi et al. explored the fecal microflora composition in piglets fed two different *L. salivarius* strains during the lactation period [94]. Specifically, significantly fewer OTUs and a lower phylogenetic diversity index and Chao index of bacteria were observed after supplementation with *L. salivarius* from normal piglets compared to the control group, suggesting that probiotics may inhibit bacterial growth. Similarly to these results, Riboulet-Bisso et al. stated that the application of *L. salivarius* UCC118 wild type reduced the number of Gram-negative bacteria present in the intestine of pigs [95]. In addition, *L. salivarius* 144 (from fast-growing pigs)-treated piglets showed a significant increase in the abundance of beneficial bacteria and a decrease in the abundance of *C. perfringens*, which may be related to their antimicrobial activity, and these changes in microbial communities may reduce the susceptibility of weaned piglets to pathogenic infections at weaning. Furthermore, *L. salivarius* (LS144 and LS160) supplementation could promote the growth of villus in all of the intestinal segments. On the contrary, in a recent study, Wei et al. found that the ingestion of *L. salivarius* WZ1 corrected the reduced species abundance and species diversity of the intestinal flora caused by *E. coli* K88 infection and increased the abundance of beneficial bacteria (*Lactobacillus* and *Bifidobacterium*), as well as decreased the abundance of harmful bacteria (*Ralstonia* and *Helicobacter*) [41]. Similar results were obtained by Xin et al. who found that the Shannon, Chao1 and Ace indices of the *Sinocyclocheilus grahami* gut microbiome were all found to increase after feeding supplemented with *L. salivarius* S01, suggesting that *L. salivarius* S01 can promote gut microbial diversity and abundance in the host [83]. These results show that the probiotic function of different *L. salivarius* strains also varies greatly.

Xu et al. found that the supplementation of *L. salivarius* CML352 in late phase improved the gut microflora composition of laying hens [88]. Specifically, this strain significantly reduced the relative abundance of the phylum Firmicutes and increased the relative abundance of the phylum Bacteroidetes, thus resulting in a significant decrease in F/B ratio. Many human studies have consistently shown that the F/B ratio is positively correlated with the degree of obesity [96]. Moreover, the gene expression levels related to methanogenesis from acetate in the *L. salivarius* CML352 group were significantly lower than in the control group, which has been presumed to reduce fat deposition and obesity [97]. Thus, *L. salivarius* strains may have the potential to reduce obesity. Additionally, the increased abundance of Bacteroidetes may promote gut health in chickens. Simply speaking, the majority of the species in the Bacteroidetes phylum are producers of SCFAs [98], which play a significant role in innate immunity, are an important source of energy for IEC, and maintain epithelial barrier function and inhibit pro-inflammatory cytokines. In addition, eggshell strength and thickness increased significantly (*p* < 0.05) after feeding *L. salivarius* CML352. This may be due to the fact that feeding *L. salivarius* CML352 increases the vitamin content of the gut and promotes the absorption of mineral elements [99]. 

For their part, Lv et al. investigated the influence of five LAB on dysbiosis produced in a rat model with acute liver injury induced by D-galactosamine [19]. They discovered that *L. salivarius* LI01 and *Pediococcus pentosaceus* LI05 were beneficial in preventing acute liver failure. Specifically, *L. salivarius* LI01 significantly decreased alanine aminotransferase and aspartate aminotransferase levels, inhibited total bilirubin accumulation, reduced the histological abnormalities of both the liver and the terminal ileum, prevented bacterial translocation, increased the serum interleukin 10 (IL-10) and/or interferon-γ (IFN-γ) levels, and resulted in a cecal microbiome that differed from that of the liver injury control. In addition, *L. salivarius* LI01 also attenuated liver fibrosis by increasing microbial abundance, improving the integrity of the intestinal barrier, and reducing plasma endotoxin levels and regulating Toll-like receptors (TLR) gene expression, among other mechanisms [90]. Furthermore, it also synergized with *Bifidobacterium* to significantly improve the symptoms of D-galactosamine-induced liver failure in rats [91]. It can be seen that in the complex OGT environment, the regulation of intestinal flora may be attributed to the synergistic effect of multiple probiotics. In conclusion, the above studies provide strong evidence for the prevention and treatment of liver injury and demonstrate that gut microbiome homeostasis contributes to the improvement of liver disease.

## 3. Multi-Omics Approach to Understanding the Role of *Ligilactobacillus salivarius*

**Figure 4:**
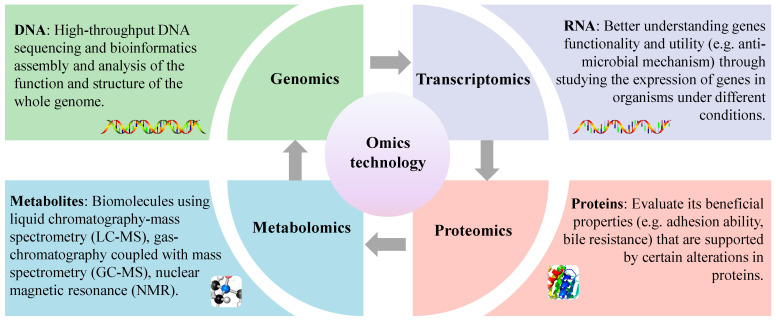
Understanding probiotic functional properties using an omics approach.

Omics technology belongs to the concept of systems biology, mainly including genomics, transcriptomics, proteomics and metabolomics, etc. With the continuous improvement and evolution of omics technology research, combined with chemometric tools, people have a comprehensive and in-depth understanding of the mechanism behind the functional and specific interactions between probiotics and hosts (Figure 4). Researchers have successfully revealed the genetic information of different microorganisms with various phenotypes using these methods. Therefore, these are very useful tools to bridge the gap between genetic information and cell-specific metabolites. Among these, genomics allows for the identification of functional genes contained in the target strain and transcriptomics, proteomics and metabolomics allow for the quantification of mRNA, proteins and metabolites (<1500 Daltons), respectively, under specific physiological conditions. Therefore, it is worth noting that all of these methods are completely different from traditional characterization methods. Considering the complexity and uniqueness of organisms, a single method is not enough to characterize organisms. Most lactobacillus has been widely used in food and pharmaceutical fields. It is only recently, however, that advances in technology and methods have revealed the mechanisms that explain the beneficial effects of these bacteria on the host [100]. To date, many scholars have integrated various “omics” approaches to understanding the functional role of LAB, including *L. salivarius* [51].

### 3.1. Genomics Approach to Comprehend the Role of Ligilactobacillus salivarius

With the rise of genomics, LAB have important research value for human production and life, and gradually become the focus of research. It is expected that this will reveal the diversity and evolution of LAB at the molecular level, enable analysis of their physiological and metabolic mechanisms, and uncover functional genes related to important traits in order to accelerate the breeding and transformation of excellent strains, and provide a basis for the efficient utilization of LAB and improvement in the industrial-level control of fermentation.

Based on the genome database of NCBI (https://www.ncbi.nlm.nih.gov/genome, accessed on 11 May 2023), United States, 22 complete genome maps of *L. salivarius* have been constructed as of May 2023. Yang et al. determined the whole genome of *L. salivarius* AR809 and it contains 1967 genes, of which a total of 1593 genes encoding proteins, 79 tRNA genes and 22 rRNA genes were found on the circular chromosome, and 240, 28, 3 and 2 genes encoding proteins were found on plasmid pA-pD, respectively [42]. In addition, genomic analysis also screened a series of genes related to beneficial properties, such as carbohydrate metabolism, environmental stress and adhesion [42], which provide valuable guidance for the oral colonization of *L. salivarius* AR809. Sun et al. reported the whole genome sequence of *L. salivarius* Ren, which contained a 1,751,565 bp circular chromosome and two plasmids [101]. Bioinformatics analysis identified several genes important for gastrointestinal tolerance and adhesion, such as genes involved in acid and bile salt tolerance responses, genes encoding S-adenosylmethionine synthase, and fibronectin-binding protein genes [101]. In addition, it found that the absence of an α-glycerophosphate oxidase coding gene in *L. salivarius* ATCC11741, which fails to degrade 4-hydroxyaminoquinoline 1-oxide (4-HAQO), suggested it may be one of the reasons for the significant differences between *L. salivarius* Ren and ATCC11741 in the secretion of H_2_O_2_ and degradation of 4-HAQO [101].

### 3.2. Transcriptomics Approach to Comprehend the Role of Ligilactobacillus salivarius

Microarray and RNA sequencing (RNA-Seq) are two main techniques currently used for transcriptomic research [102]. In the probiotics field, the transcriptome has been used to study the molecular mechanisms involved in environmental stress responses, e.g., tolerance to the stress of acid and bile salts in the GIT [51]. In addition, transcriptomics help to reveal molecular strategies for probiotic interactions with the host or other microbiota, including changes in metabolic profiles, signaling pathways regulation, cell growth and communication (intercellular signaling) [103]. Song et al. used RNA sequencing analysis to identify the first well-characterized endogenous constitutive promoter library from *L. salivarius*, providing a useful toolbox for the subsequent metabolic engineering and synthetic biology of *L. salivarius* and other prokaryotes [104]. Xia et al. fused a multi-omics strategy of transcriptomics, metabolomics and cytokine arrays to explore the effects of colonization with *L. salivarius* LI01 on growth, immunity and metabolism in germ-free rats [105]. Similarly, Lv et al. used transcriptome sequencing combined with proteome, and proposed the first model of a bile stress response mechanism for *L. salivarius* LI01, which provides a reference for the subsequent bile salt resistance mechanism of *L. salivarius* [51]. The combination of transcriptomics and other omics is one of the most common methods to solve experimental problems. Considering the specific environment, the integrated study of transcriptome combined with other technologies can be used in many fields, e.g., studying the role of LAB in food spoilage, potential probiotic properties of LAB strains, etc.

### 3.3. Proteomics Approach to Comprehend the Role of Ligilactobacillus salivarius

Proteomics mainly studies the dynamic changes in proteins during development and their responses to internal or external stimuli [106]. Based on the complexity of protein structure, researchers have developed a comprehensive proteomics technique to deeply analyze all of the proteins present in a sample [107]. In recent years, the proteomics research on *L. salivarius* has been increasing (Table 3). Kang et al. used 2D gel electrophoresis and matrix-assisted laser desorption/ionization time-of-flight/time-of-flight mass spectrometry (MALDI-ToF/ToF MS) analysis and identified potential secreted proteins that may be responsible for the antimicrobial activity [108]. A total of 21 secreted proteins were identified, of which five were produced by *L. salivarius*. The LysM domain protein was a peptidoglycan binding protein that may cause the lysis of *S. aureus* upon binding to the cell wall but does not affect lactobacillus. Kelly et al. identified three proteins, DnaK, Ef-Ts and pyruvate kinase, in the cell wall of *L. salivarius* UCC118 by combining proteomics analysis with enzymatic techniques [109]. These proteins may play an important role in adhesion and promoting host immune perception. In addition, the proteome can also be used for strain identification. Hamza et al. used MALDI-TOF spectroscopy and 16S rDNA sequencing to identify 67 isolates [110]. All identified isolates were *L. salivarius* and *L. plantarum*. Proteomic analysis can help to understand the different characteristics of *L. salivarius* and identify the proteomic profile of individual characteristics, which can be used as biomarkers for the initial selection of potential probiotic strains.

### 3.4. Metabolomics Approach to Comprehend the Role of Ligilactobacillus salivarius

The metabolome is believed to be the result of the genome, transcriptome and proteome, and directly influences the molecular phenotype of microbial cells. Its application in probiotics research has been developed rapidly in the past few years. Metabolomics has been used to map metabolic pathways and reveal microbial metabolic networks, e.g., studying types and changes of metabolites in food, evaluating the effect of probiotics on the metabolic activity of resident microflora and characterizing microbial molecules secreted during the industrial production of probiotics. Zhu et al. used genomics and metabolomics to explore the alleviation mechanism of the *L. salivarius* strain on nonalcoholic fatty liver disease (NAFLD) [112]. Specifically, most of the 250 metabolites from *L. salivarius* SNK-6 are involved in multiple metabolic pathways, including amino acid and lipid metabolism. Studies have reported that the *L. salivarius* strain can effectively alleviate liver injury by regulating liver lipid metabolism. In addition, a further analysis of the metabolomics data showed that butyric acid, acetic acid and propionic acid were the main SCFAs produced by *L. salivarius* strain, while cholic acid (CA), ursodeoxycholic acid (UDCA), chenodeoxycholic acid (CDCA) and tauroursodeoxycholic acid (TCA) were the four most abundant bile acids in the metabolites of *L. salivarius* SNK-6. Studies have shown that they are important signaling molecules that regulate lipid metabolism genes and can alleviate lipid accumulation and inflammation in NAFLD rats. Bile acids play an important role in regulating lipid, glucose and energy metabolism, and are essential for protecting hepatocytes from cholesterol and bile acid toxicity. CA shows a specific affinity for bile acid receptors, and as a signal molecule, it has a unique role in regulating liver lipid metabolism. UDCA is used to treat primary biliary cirrhosis, activate PKC and MAPK signaling and anti-inflammatory hepatocyte pathways, and promote bile HCO_3_^−^ secretion to reduce cholestasis and liver damage. CDCA can reduce body weight and improve glucose tolerance. TCA can reduce hepatic steatosis, intestinal inflammation and insulin resistance in mice. 

In summary, we found that genomics and transcriptomics can analyze the genetic information and functional genes of *L. salivarius*, and proteomics and metabolomics can delve into the adaptive mechanism of *L. salivarius* in response to physiological and environmental changes. Therefore, when we study *L. salivarius*, the combined use of the above four histological approaches can comprehensively reveal the complex mechanisms behind the functions and beneficial properties of *L. salivarius* from multiple perspectives, including genetics, expression and metabolism.

## 4. Conclusions

In recent years, *L. salivarius* has gained increasing attention from researchers due to its tolerance to the oral cavity and gastrointestinal environment, as well as its ability to adhere to human cells. Studies have shown that *L. salivarius* strains have various physiological functions, such as anti-oxidation, immune regulation, and maintaining the balance of intestinal flora, providing a theoretical and experimental basis for the development of functional lactic acid bacteria products and healthy foods with the potential to lower blood fat and prevent cardiovascular diseases. Despite the recognized functional properties of different *L. salivarius* strains, further research is needed to fully understand the role this microbe plays in host health. Omics approaches are becoming increasingly important in this field of research as they enhance the understanding of the functions and mechanisms behind the probiotic properties of beneficial bacteria such as *L. salivarius*. Genomics and transcriptomics can reveal the genetic information of *L. salivarius* strains, analyze their physiological and metabolic mechanisms, and discover important functional genes. Proteomics and metabolomics can study the effect of *L. salivarius* strains on the mechanisms of adaptation to physiological and environmental changes, such as adhesion, biofilm formation, antagonistic capacity, and tolerance. However, there are still many questions that need to be clarified, emphasizing the importance of further research in the field of multi-omics techniques to increase our understanding of how *L. salivarius* exerts its probiotic activity.

## Figures and Tables

**Table 1:** Probiotic properties and applications of *Ligilactobacillus salivarius*.

Strain and Origin	Probiotic Properties	Potential Application	Reference
F14	Good tolerance to acid (pH 3.0). Resistance to 0.3% bile salt. Antimicrobial activity against *Pseudomonas aeruginosa*, *S. aureus*, *Salmonella typhimurium* and *K. pneumoniae*. Adhesion to Caco-2 cells.	Foodborne illnesses outbreaks	[10]
CGMCC20700	Resistance to acid (pH 2.0) and 0.9% bile salt. Good auto-aggregation (57.12%) and hydrophobic (61.16%) capability. Antimicrobial activity against Gram-positive and -negative bacteria.	Livestock and poultry farming	[11]
Eleven isolates	Good resistance to acid and bile salt. Good auto-aggregation and co-aggregation potential. Antimicrobial activity against *Escherichia coli* and *Salmonella arizonae*. Adhesion to HT-29 cells. Has antioxidant potential. Produces digestive enzymes, e.g., amylase, protease and β-galactosidase.	Animal probiotics	[12]
CECT 30632 (MP101)	Resistance to gastrointestinal conditions. Adhesion to both vaginal and intestinal cells. Produces α-amylase.	Habitual abortion/Infertility	[13]
PS21603	Resistance to osmotic changes and acid (pH 2.0). High antimicrobial activity.	Pig diarrhoea	[14]
UCC118 (human ileal-ceca region)	Resistance to acid and bile. Good adhesion to HT-29 and Caco-2 cells. Produces a small heat-stable bacteriocin Abp-118 and has a broad-spectrum antimicrobial activity.	Bacterial infection	[15]
Ren (human feces)	Good resistance to gastric acid, intestinal fluid and bile. High affinity to intestinal mucus and epithelial cells. Effectively inhibited the growth of *Helicobacter* strain. Supplementation of *L. salivarius* Ren or its metabolites could effectively inhibit oral and colon cancer.	Colon cancer/Oral cancer	[16,17,18]
Li01 (healthy human)	Strong antimicrobial activity against enteropathogens. Tolerance to acid and bile stress. Had good effects in the prevention and treatment of liver failure.	Liver disorders	[19]
CECT5713 (human breast milk)	Good gastrointestinal tolerance and good adhesion to both Caco-2 and HT-29 cells. Has antimicrobial activity. Produces L-lactate, acetate and H_2_O_2_, does not produce biogenic amines. Enhances both natural and acquired immune responses.	Bacterial infections/Inflammatory bowel diseases/Lactational mastitis/Dental caries/Infantile formula	[20,21]
BP121 (infant feces)	High survival rate in gastrointestinal tract (GIT). Has antimicrobial activity. Prevents cisplatin-induced acute kidney injury (AKI).	Acute kidney injury	[22]
E4191 (infant feces)	70% adhesion to HT-29 cells. Exhibits anti-inflammatory effects through repressing IL-8 mRNA expression.	Inflammatory bowel diseases	[23]
FFIG strains (intestine of pigs)	Has adhesion capacity to porcine mucins and porcine intestinal epithelial (PIE). Functionally modulates the innate immune responses triggered by TLR3 and TLR4 activation in PIE cells and effectively adheres to these cells. Reduction of rotavirus replication levels in PIE cells.	Bacterial infections in pigs	[24]
Ls-33	Tolerance to bile salts. Good tolerance to pancreatin. Good adhesion to Caco-2 cells compared with *Lactiplantibacillus plantarum* NCIMB8826, *L. plantarum* Lp-115 and *Lactobacillus acidophilus* NCFM. Supplementation of *L. salivarius* Ls-33 significantly prevents colitis in mice.	Inflammatory bowel diseases	[25]
FXJXJ7-2 (human feces)	Higher tolerance to GIT conditions compared with *L. salivarius* HN26-4 and NT4-8. Has a significant anti-inflammatory effect in LPS-treated RAW264.7 murine macrophages.	Inflammatory bowel diseases	[8]
PS2 (Human breast milk)	Possesses potent antibacterial activities. Effectively prevents mastitis.	Lactational mastitis	[26]
TUCO-L2 (Lama glama milk)	Good resistance to bile salt and NaCl. Good auto-aggregation and co-aggregation. Antimicrobial activity against Gram-negative intestinal pathogens. Good adhesion to mucins and PIE.	Intestinal infections	[27]
PS7 (human breast milk)	Displays strong antimicrobial activity against otopathogens. Good tolerance to GIT conditions. High adhesion to Caco-2 and HT-29 cells. Produces bacteriocin, L-lactate and acetate, does not produce H_2_O_2_ and D-lactate.	Acute otitis media	[28]
Probio-37 (Porcine gastro-intestinal tract)	Antibacterial and antiviral activities. Resistance to gastric juice and 5% porcine bile. Has a broad-spectrum antibiotic resistance.	Intestinal infections in piglets	[29]
CTC2197 (chicks’ gastro-intestinal tract)	High degree of adhesiveness to chicken intestinal epithelial cells (IEC). Antagonistic activity against *Salmonella enteritidis* and *E. coli*. Tolerance to bile salts and acid (pH 3.0).	Foodborne salmonellosisoutbreaks	[30]
UO.C003/UO.C027 (chicken ceca mucosa)	Antagonistic activity against *Salmonella*, *Clostridium*, and *E. coli*. Good resistance to acidic pH. Adhesion to IEC.	Foodborne illnessesoutbreaks	[31]
FP25/FP35 (healthy infant feces)	Antagonistic activity against enteric pathogens. Good resistance to acid (pH 2.5) and 0.3% bile salt. High adhesion to colon cancer cells. Produces SCFAs.	Colon cancer	[32]
MM1 (healthy infant feces)	Antagonistic activity against enterotoxigenic *E. coli* (ETEC). Tolerance to acid (pH 2.0) and 0.3% bile salt. Adhesion to Caco-2 cells.	Diarrheal illness	[33]
AR809 (human pharynx)	Antimicrobial activity against adhesion to *S. aureus*. Tolerance to acid, bile, lysozyme and H_2_O_2_. Adhesion to FaDu cells. Alleviating pharyngitis injury in rats.	Pharyngitis	[34,35]
MG4265 (human)	Good antagonistic activity against *Streptococcus mutans*. Actively suppresses RANKL-induced osteoclast genesis.	Periodontitis/dental caries	[36]
NK02 (oral cavity)	Good tolerance to gastric juice, lysozyme and bile. Antagonistic activity. Tolerance to hydrolyze sodium salt of glycodeoxycholic acid, tolerates 1–5% of NaCl.	Periodontitis	[37]
MG242 (human vagina)	Antagonistic activity against *Gardnerella vaginalis* and *Candida albicans*. Tolerance to pancreatin, pepsin and acidic environment.	Vulvovaginal candidiasis	[38]
CRL1328 (human vagina)	Antagonistic activity against genitouropathogenic *S. aureus*. Adhesion to vaginal epithelial cells.	Urogenital tract infections	[39]
UCM572 (human vagina)	Adhesion to bladder epithelial cells. Antagonistic activity.	Urinary tract infections	[40]
WZ1	Resistance to acidic condition and bile salts. Has a good inhibitory effect on pathogens, such as *E. coli*, *Salmonella*, *S. aureus* and *C. perfringens*.	Preventing diarrhea	[41]

**Table 2:** Effect of different *Ligilactobacillus salivarius* strains on the intestinal microbiota.

*L. salivarius* Strains	Main Effects on Gut Microbiota	Reference
S01	Reduced the presence of Proteobacteria phylum. Increased Firmicutes, Bacteroidota and Actinobacteriota phylum. Intestinal microflora was decreased and overall microbial diversity increased. Reduced *Aeromonas* and increased *Bifidobacterium*.	[83]
CGMCC 1.1881	Relative abundance of *L. salivarius* and *Ligilactobacillus agilis* were significantly improved in the co-feeding group of *L. salivarius* + *L. agilis* and *L. salivarius* + *Ligilactobacillus aviaries*. Relative abundance of *E. coli* was significantly decreased in the *L. salivarius* + *L. agilis* group.	[84]
CPU-01	Restored temozolomide-induced reduction in bacterial abundance and diversity. Decreased the abundance of *Campylobacterota*, *Deferribacterota*, and *Verrucomicrobiota*. Increased the abundance of Bacteroidota and Firmicutes, and increased the ratio of Firmicutes/Bacteroidota (F/B ratio). Increased the abundance of *Lactobacillaceae*, *Lactobacillales*, *Ligilactobacillus* and *Chitinophagales*.	[50]
SMXD51	Partially limited the effects of *Campylobacter* on *Anaerotruncus* sp. decrease and *Subdoligranulum* sp. increase. Decreased the abundance of *Faecalibacterium*. Significantly enriched *Escherichia* sp. and *Flavonifractor* sp.	[85]
CI1, CI2 and CI3 mix	Increased the abundance of beneficial bacteria such as *Lactobacilli* and *Bifidobacteria*. Decreased the abundance of harmful bacteria such as *E. coli* and total aerobes. Reduced harmful cecal bacterial enzymes.	[86]
*L. salivarius*	Increased the number of *Lactobacillus* sp. Decreased plasma ammonia content, the number of *E. coli* in ceca and fecal ammonia emission.	[87]
CML352	Reduced Firmicutes and increased Bacteroidetes phylum. Reduced the metabolisms of lipids and acetate. Enlarged the number of SCFA-producing bacteria. Increased the relative abundance of the microbes involved in GDP-mannose biosynthesis.	[88]
SNK-6	Increased the abundance of Firmicutes. Decreased the abundance of Bacteroidetes.	[89]
LI01	Altered the cecal microbiome composition. Prevented bacterial translocation in liver injury.	[19]
LI01	Alleviated the dysbiosis of intestinal microflora. Increased the F/B ratio. Increased the relative abundance of *Prevotella*, *Paraprevotella*, *Lactobacillus* and *Elusimicrobium*. Decreased bacterial species related to pro-inflammatory cytokines and profibrogenic genes. Increased the gut microbiota diversity.	[90]
LI01	Higher abundance of *Alloprevotella*. Reduced in *Ruminococcus*_1, *Prevotellaceae*_NK3B31_group and *Eubacterium_xylanophilum*_group. Depleted *Prevotellaceae*_NK3B31_group, *Roseburia* and *Lachnospiraceae*_UCG-001 with the coadministration of *L. salivarius* LI01 and *B. longum* TC01.	[91]
CECT5713	Increased the number of faecal *Lactobacilli*. Maintained the dynamic balance of *Bifidobacterium*. Did not alter the *Bacteroides*, *Clostridia*, total aerobes or *Enterobacteria*.	[92]
HN26-4, NT4-8 and FXJCJ7-2	Altered gut microbiota structure. Increased the relative abundance of *Allobaculum*, *Lactobacillus*, *Streptococcus*, *Veillonella* and *Ruminococcus*. Enlarged SCFA-producing bacteria. Reversed the decrease in acetic acid, isobutyric acid, valeric acid and total SCFA levels caused by LPS.	[8]
HK LS 189	Changed the gut microbiota diversity. Decreased the relative abundance of *Prevotella*, *Blautia*, *Lachnospira*, *Mitsuokella* and *Anaerostipes*. Regulated the metabolic functions of the intestinal microflora. Increased the metabolisms of lipids.	[93]
WZ1	Corrected the reduced species abundance and species diversity of the intestinal flora caused by *E. coli* K88 infection. Improved the levels of beneficial bacteria such as *Lactobacillus* and *Bifidobacterium*. Reduced the levels of harmful bacteria such as *Ralstonia* and *Helicobacter*.	[41]

**Table 3:** Potential functional properties of *Ligilactobacillus salivarius* revealed using omics techniques.

Applied Omic Technologies	Total Number of Identified Genes/ Compounds	Functional Properties Revealed with Omic Approaches	Reference
Transcriptomics			
Digital Gene Expression (DGE)	2014	Helping the cells to maintain surface homeostasis. Enhancing the hydrophobicity of the cell surface. May be helpful for tolerating bile exposure. May help to promote attachment of secondary cell wall polymers with discrete linkage units to peptidoglycan, maintaining the structure of the cell wall. The bile reaction mechanism was studied for the first time in *L. salivarius* using transcriptomics.	[51]
RT-qPCR and microarray assay	ND	Feasibility of finding specific molecules as new targets responsive to oral *L. salivarius* PS2 in mastitis-infected human mammary glands.	[111]
RNA-sequencing and RT-qPCR	2112	Native constitutive promoters with various strengths were identified in *L. salivarius*.	[104]
High-throughput transcriptomic sequencing and qRT-PCR	Not specified	Upon *L. salivarius* + *L. agilis* feeding, in crypt, genes involved in oxidative phosphorylation, biosynthesis of amino acids, and ribosome-related pathways were activated.	[84]
RNA-sequencing and RT-qPCR	>1941	Stimulated the immune response and metabolic process by altering transcriptional expression in the ileum and liver.	[105]
RNA-sequencing and RT-qPCR	1990	Response to bile stress.	[60]
Proteomics			
iTRAQ LC-MS/MS	1260	The bile reaction mechanism was studied by proteomics in *L. salivarius* for the first time.	[51]
2D, CapLC-MS/MS	20	Identification of proteins related to intestinal mucosal adhesion (DnaK, EF-Ts and Pyruvate kinase).	[109]
2DE, MALDI-TOF-MS	21	Antibacterial activity.	[108]
MALDI-TOF-MS	67	Molecular identification of the most relevant *Lactobacillus* strains.	[110]
2DE, MALDI-TOF-MS	34	Response to bile stress by up-regulating proteins related to amino acid transport metabolism, carbohydrate transport metabolism and redox homeostasis.	[60]
Metabolomics			
UHPLC-MS/MS	250	Acetate derived from *L. salivarius* SNK-6 mediates the interaction between *L. salivarius* SNK-6 and liver metabolism. Bile acids (BA) are crucial to protect liver cells from cholesterol and bile acid toxicity.	[112]
^1^H-NMR	7 significant metabolites	Probiotic supplementation decreased lactose excretion which could suggest a normalization of breast permeability. Increased levels of TMAO, creatine and hippurate might suggest changes in choline metabolism, in energetic pathways and in gut microbiota metabolism.	[113]
UHPLC-MS/MS, GC-MS	ND	SCFAs, particularly butyrate, have the effect of stimulating tight junction and mucous production, reducing systemic inflammation and regulating intestinal hormones. BA metabolism was related to BA biosynthesis in the liver and biotransformation by gut microflora.	[114]
UHPLC-MS/MS	381	Succinate transported to ISCs, combined with MMP and ROS levels, can promote ISC activity.	[115]
^1^H-NMR	44	These metabolites are directly involved in the major biochemical reactions and key metabolic pathways associated with cellular osmotic stress response.	[116]
LC-MS/MS	109	Identified compounds had potential antimicrobial activities. Addition of probiotics enriched some potentially beneficial microbes and small molecular compounds with antimicrobial activity, and inhibited potential pathogens in fermented feed.	[117]
^1^H-NMR	More than 50 molecules	Possible presence of bacteriocins in the identified amino acids, resulting in antimicrobial activity of the supernatant.	[118]
LC-MS/MS	778	Compounds related to tryptophan metabolism and primary BA biosynthesis were identified.	[84]
Untargeted GC-MS	348	Modulated carbohydrate metabolism and arginine transaction.	[105]

## Data Availability

No new data were created or analyzed in this study. Data sharing is not applicable to this article.
